# Supplementary material for: Carbon dioxide insufflation reduces the relapse of ulcerative colitis after colonoscopy: A randomized controlled trial
Source: PLoS One. 2023 Aug 17;18(8):e0290329. doi: 10.1371/journal.pone.0290329 (PMC10434883; doi:10.1371/journal.pone.0290329)
Supplement: S1 Text — (DOCX) [file pone.0290329.s002.docx]

Protocol Title

Evaluation of colonoscopy with carbon dioxide insufflation

in patients with ulcerative colitis: a randomized controlled trial

Protocol of the study

A randomized controlled trial to evaluate the effects of colonoscopy with carbon dioxide insufflation on recurrence in patients with ulcerative colitis

I. Background and purpose of the study

1. Background and objectives

For patients with ulcerative colitis (UC), colonoscopy is not only essential for diagnosing but also useful for evaluating treatment efficacy because mucosal healing is a recently established therapeutic goal. In addition, patients with long-term diseases are at high risk for colitic cancer and require lifelong periodic colonoscopies for cancer surveillance. However, clinical symptoms are often exacerbated after colonoscopy, and a previous study showed that 16% of patients with UC experienced an aggravation of their clinical symptoms (Menees S et al. [Inflamm Bowel Dis.](http://www.ncbi.nlm.nih.gov/pubmed/17206634), 2007).

A recent study reported the safety of colonoscopy using carbon dioxide (CO_2_) insufflation, which reduced subjective symptoms of patients after examination, compared with colonoscopy using air insufflation (Wu J et al. Endoscopy. 2012), and the use of CO_2_ insufflation in clinical practice is increasing. However, very few reports have investigated the effect of CO_2_ insufflation during colonoscopy on the clinical course of patients with UC. Demonstrating the beneficial effects of colonoscopy using CO_2_ insufflation compared with air insufflation to reduce the relapse rate of UC will lead to the establishment of a standardized test method.

II. Study subjects

1. Number of cases and the rationale for setting the number of the cases

(1) Number of cases

250 cases

(2) Basis for setting

Assuming that the exacerbation rate in the air insufflation group is 16% and that in the CO_2_ group is 5%, and that the alpha error and power are 0.05 and 0.8, respectively, 119 patients are required for each group—238 patients are required for both groups combined. The number of patients with UC attending our hospital is approximately 250, and we assumed that we could gather a large number of cases in 2 years.

2. Selection criteria

Patients with UC in clinical remission (partial Mayo score [PMS] ≤ 2) who are scheduled to undergo colonoscopy at Osaka University Hospital and who provide sufficient informed consent.

3. Exclusion criteria

(1) Patients with a history of colorectal resection

(2) Patients in clinically active phase (PMS ≥ 3)

(3) Patients on complete intravenous nutrition

(4) Patients with chronic obstructive pulmonary disease

(5) Patients with heart failure

(6) Patients under 15 years old

(7) Patients undergoing sedated colonoscopy

(8) Patients in extremely poor general condition

(9) Patients deemed inappropriate by the physician for other reasons

III. Study method

1. Study design

Single-center, prospective, single-blind, randomized controlled trial

2. Registration and allocation method

Patients with UC who do not meet the exclusion criteria will be randomly enrolled in (1) the CO_2_ insufflation group and (2) the air insufflation group.

3. Outline of the examination

(1) The study will be explained orally and in writing, and consent will be obtained in writing. For patients under 20 years of age, consent will be obtained from the patient and a legal representative.

(2) Patients whose consent is obtained will be randomly assigned to the CO_2_ or air insufflation groups (1:1).

(3) Patients will not be told which group they are assigned to and will undergo colonoscopy. Pretreatment will be performed with polyethylene glycol (Moven®) or sodium phosphate (VISICLEAR®) only. The endoscope that will be used is the CF-H260AZI or CF-HQ290ZI, manufactured by OLYMPUS. Moreover, a change in the endoscope used during the examination due to stenosis or poor operability is acceptable. Before and after the examination, the patients will be interviewed regarding their symptoms and physical findings.

(4) Patients will be examined at 1 and 8 weeks after the colonoscopy, and the clinical course will be investigated based on blood test results, symptoms, physical findings, and treatment details.

4. Observations and survey items

(1) Patient background

Sex, age, concomitant medications, smoking history, duration of disease (from diagnosis), and disease type (extent of disease and clinical course) will be investigated.

(2) Subjective symptoms

Abdominal fullness, abdominal pain, defecation and bloody stool frequencies, and general condition before and after colonoscopy will be assessed.

(3) Endoscopy

Endoscopic findings will be evaluated based on the Mayo endoscopic subscore. Time to reach the cecum, examination time, percentage of cecum intubation, blood pressure, and SpO_2_ before and after endoscopy will be measured, and the number of years of endoscopic experience of the physician in charge of the examination will be recorded.

(4) Blood test

White blood cell count, hemoglobin, hematocrit, platelet count, C-reactive protein, and albumin will be measured.

| Schedule | | | | | |
| --- | --- | --- | --- | --- | --- |
| Item | | Pre-observation period | Colonoscopy | Post-colonoscopy examination | |
| Period | | 0–8 weeks before colonoscopy | 0 weeks | Examination  after 1 (~2) week(s) | Examination  after 8 (±2) weeks |
| Seeing a doctor | | Seeing a doctor 1 |  | Seeing a doctor 2 | Seeing a doctor 3 |
| Obtaining consent | | ● |  |  |  |
| Allocation | | ● |  |  |  |
| Subjective symptoms and other findings | | ● |  | ● | ● |
| Blood pressure/pulse measurement | | 〇 | ● | 〇 | 〇 |
| Concomitant medications | | ● | 〇 | ● | ● |
| Clinical examination | Hematologic examination | ● | 〇 | ● | ● |
|  | Blood biochemical test | ● | 〇 | ● | ● |
| Colonoscopy | |  | ● |  |  |
| Partial Mayo  Score | | ● | 〇 | ● | ● |
| Medical questionnaire | |  | ● | ● | ● |

●: Required

〇: Obtained if possible

5. Analysis method

After linkage-anonymization, the images, patient background, subjective symptoms, and laboratory data will be analyzed at the Department of Gastroenterology and Hepatology, Osaka University.

6. Response to adverse events

In the case of an adverse event*, the patient will be informed, and appropriate action will be taken promptly under insurance. When the principal investigator becomes aware of a serious adverse event or failure incidence related to clinical research, he/she will immediately notify the head of the clinical research institute.

*Adverse events are defined as those events deemed by the physician to be harmful regardless of whether or not they are causally related to the protocol treatment, and the following are treated as serious adverse events

a. Events resulting in death

b. Life-threatening occurrence

c. Events that can result in permanent or marked disability or dysfunction.

7. Evaluation items

(1) Primary endpoint

- The clinical recurrence rate at 1 week after colonoscopy

(2) Secondary endpoints

- The clinical recurrence rate at 8 weeks after colonoscopy and changes in treatment
- Pre- and post-colonoscopy vital signs (blood pressure, SpO_2_), subjective symptoms (abdominal pain, bloating)
- Cecum intubation rate, cecum intubation time, and total examination time

8. Statistical analysis

- Contingency table analysis: chi-square test, Fisher's exact test
- Comparison of two groups without correspondence: Mann–Whitney U test
- Multi-group comparison: Steel–Dwass test and Tukey–Kramer HSD test, among others.

9. Anticipated medical contributions

While colonoscopy is essential for patients with UC for diagnosis, disease monitoring, evaluation of treatment efficacy, and cancer surveillance, clinical symptoms may worsen after colonoscopy. If the usefulness of CO_2_ insufflation colonoscopy for patients with UC is scientifically demonstrated, it will establish a less invasive and more appropriate examination method for patients with UC.

10. Study discontinuation criteria

(1) When a patient requests discontinuation of the study after it has started

(2) When it is judged to be extremely difficult to achieve the planned number of cases due to difficulties in recruiting participants

(3) If the objective of the study is achieved (e.g., by interim analysis) before the expected number of patients or the expected duration of the study is reached

(4) When there is a recommendation or instruction for discontinuation by the Clinical Research Ethics Review Committee

(5) Other cases in which the investigator determines that the study should be terminated

11. End of the examination

At the end of the study, the investigator shall promptly submit an End of Study Report to the hospital.

12. Study period

The study will be from 2015, after Study Approval, to December 31, 2019.

IV. Medical ethical considerations

1. How to obtain consent

The study will be explained orally and in the attached patient information sheet, and consent will be obtained in writing. The consent will be obtained after stating in the explanation document that participation in the study is voluntary, no disadvantages will be incurred if the patient does not agree to participate, and the patient has the right to withdraw the consent obtained for the study at any time without any disadvantages. For minor patients, consent will be obtained from the patients themselves and their parents or guardians.

2. Consideration for human rights

This clinical research will be conducted in compliance with the Ethical Guidelines for Clinical Research (Ministry of Health, Labour, and Welfare) and the Declaration of Helsinki (World Medical Association).

3. Consideration of safety disadvantages

Adverse events are defined as those events deemed by the physician to be harmful regardless of whether or not they are causally related to the protocol treatment, and the following are treated as serious adverse events.

a. Events resulting in death

b. Life-threatening incidence

c. Events that can result in permanent or marked disability or dysfunction.

In the case of a serious adverse event, the head of the facility and the principal investigator will be notified immediately.

4. Protection of personal information

When handling data related to the study, due consideration will be given to protect the confidentiality of the participants.

5. Publication of research results

After the completion of the study, data analysis will be promptly performed, and the data will be presented at a conference and submitted for publication in an academic journal.

6. Cost-sharing of testing

Because the study is within the scope of usual medical care, the health insurance of patients will be applied as usual practice. No gratuities will be paid to the patients.

7. Conflict of interest

No special funding was received for this study, and no conflicts of interest exist in the planning, conduct, or reporting of the study that would affect the results of the study or the interpretation of the results.

8. Record keeping

After confirming that no personal information is included, the materials obtained from the study will be disposed of immediately after the study is completed.

V. Participating institutions and principal investigators

Principal investigator:

Tetsuro Takehara, Professor, Department of Gastroenterology and Hepatology, Osaka University Graduate School of Medicine

Co-investigators:

Hideki Iijima, Department of Gastroenterology and Hepatology, Osaka University Graduate School of Medicine

Kenji Watabe, Center for Medical Education, Osaka University

Shinichiro Shinzaki, Department of Gastroenterology and Hepatology, Osaka University Graduate School of Medicine

Yasutoshi Nozaki, Department of Gastroenterology and Hepatology, Osaka University Graduate School of Medicine

Shunsuke Yoshii, Department of Gastroenterology and Hepatology, Osaka University Graduate School of Medicine

Yuto Shioide, Department of Gastroenterology and Hepatology, Osaka University Graduate School of Medicine

Shuko Iwatani, Department of Gastroenterology and Hepatology, Osaka University Graduate School of Medicine

Akihiko Sakatani, Department of Gastroenterology and Hepatology, Osaka University Graduate School of Medicine

Keiichi Kimura, Department of Gastroenterology and Hepatology, Osaka University Graduate School of Medicine

Takeo Yoshihara, Department of Gastroenterology and Hepatology, Osaka University Graduate School of Medicine

Kazuhiro Murai, Department of Gastroenterology and Hepatology, Osaka University Graduate School of Medicine

Katsuhiko Sato, Department of Gastroenterology and Hepatology, Osaka University Graduate School of Medicine

Takanori Inoue, Department of Gastroenterology and Hepatology, Osaka University Graduate School of Medicine

Satoshi Shigeno, Department of Gastroenterology and Hepatology, Osaka University Graduate School of Medicine

Naoki Mizutani, Department of Gastroenterology and Hepatology, Osaka University Graduate School of Medicine

Ryotaro Uema, Department of Gastroenterology and Hepatology, Osaka University Graduate School of Medicine

Yuriko Otake, Department of Gastroenterology and Hepatology, Osaka University Graduate School of Medicine

Mizuki Tani, Department of Gastroenterology and Hepatology, Osaka University Graduate School of Medicine

Yuta Myojin, Department of Gastroenterology and Hepatology, Osaka University Graduate School of Medicine

Shinnosuke Kudo, Department of Gastroenterology and Hepatology, Osaka University Graduate School of Medicine

Atsushi Maeda, Department of Gastroenterology and Hepatology, Osaka University Graduate School of Medicine

Takahiro Amano, Department of Gastroenterology and Hepatology, Osaka University Graduate School of Medicine

Minoru Kato, Department of Gastroenterology and Hepatology, Osaka University Graduate School of Medicine

Makoto Fukuoka, Department of Gastroenterology and Hepatology, Osaka University Graduate School of Medicine

Yu Sato, Department of Gastroenterology and Hepatology, Osaka University Graduate School of Medicine

Kenji Fukumoto, Department of Gastroenterology and Hepatology, Osaka University Graduate School of Medicine

Research collaborators:

Toshifumi Ito, Chief, Department of Gastroenterology, Osaka Hospital, Japan Community Healthcare Organization (JCHO)

Satoshi Hiyama, Medical Staff, Department of Gastroenterology, Osaka Hospital, Japan Community Healthcare Organization (JCHO)
