# Supplementary figures and images for: Carbon dioxide insufflation reduces the relapse of ulcerative colitis after colonoscopy: A randomized controlled trial
Source: PLoS One. 2023 Aug 17;18(8):e0290329. doi: 10.1371/journal.pone.0290329 (PMC10434883; doi:10.1371/journal.pone.0290329)

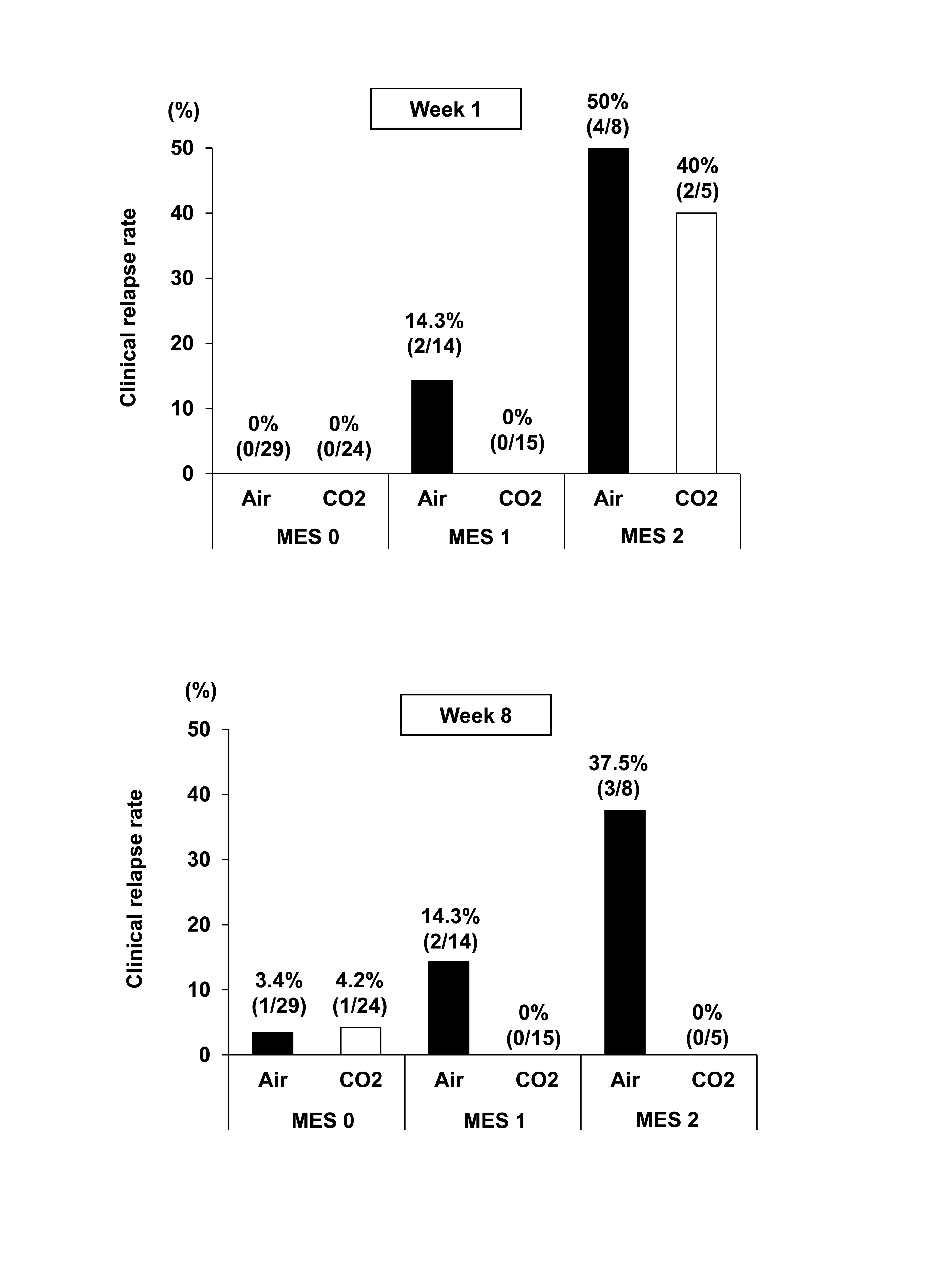

Supplement: S1 Fig — (TIF) [file pone.0290329.s003.tif]
